# Sex Differences in Concussion Rates among Middle School Sex-comparable Sports

**DOI:** 10.1016/j.jpedcp.2026.200200

**Published:** 2026-01-23

**Authors:** Samantha L. Hacherl, Daniel C. Herman, Joel R. Martin, Patricia M. Kelshaw, Sanja Avramovic, Shane V. Caswell

**Affiliations:** 1 Graduate Health Sciences, Franklin College, Franklin, IN; 2 Virginia Concussion Initiative, George Mason University, Manassas, VA; 3 Department of Physical Medicine and Rehabilitation, University of California at Davis, Davis, CA; 4 Sports Medicine Assessment Research & Testing (SMART) Laboratory, George Mason University, Manassas, VA; 5 Center for the Advancement of Well-Being, George Mason University, Fairfax, VA; 6 Department of Kinesiology, Brain Research and Assessment Initiative of New Hampshire (BRAIN), University of New Hampshire, Durham, NH; 7 Department of Health Administration and Policy College of Public Health, George Mason University, Fairfax, VA

**Keywords:** concussion, early adolescent, epidemiology, prevention, youth sports

## Abstract

**Objective:**

To investigate sex differences in sports-related concussion (SRC) rates among early adolescents participating in sex-comparable middle school (MS) sports.

**Study design:**

This prospective epidemiological study was conducted as part of the Advancing Health Care Initiatives for Underserved Students project in Virginia from 2015 to 2022. Athletic trainers collected injury and athlete exposure (AE) data from athletes MS athletes participating in sex-comparable sports, including baseball/softball, basketball, soccer, and track and field. Outcomes included SRC injury rates (IR)/1000 AE, IR ratios (IRRs) for overall sex and event-type differences (competition vs practice), and injury proportion ratios for injury mechanisms.

**Results:**

A total of 11,213 athletes (girls, n = 5647; boys, n = 5566) contributed 329,269 AEs, during which 97 SRCs were diagnosed (0.29/1000 AE). Girls were more than twice as likely as boys to sustain an SRC overall (IRR, 2.26). In 3 of the 4 sex-comparable MS sports, girls had higher SRC rates in competition and practice than boys: softball/baseball (competition/practice IRR, 4.19/2.35), basketball (3.77/2.74), and soccer (1.75/4.92). No sex differences were observed in track and field or in the proportional distribution of SRC mechanisms.

**Conclusions:**

Among early adolescents participating in sex-comparable MS sports, girls were more than twice as likely as boys to sustain a SRC. The magnitude of the sex difference in SRC rates observed among MS athletes exceeded that previously reported in high school and collegiate populations. These findings demonstrate that sex-based differences in SRC risk are evident by early adolescence and highlight the need for developmentally appropriate, female-specific concussion prevention and clinical care strategies.

Nearly 30 million school-age children participate in organized sports each year in the US.^1^ Sports-related concussions (SRCs) are a prevalent injury, with estimates indicating that between 1.1 and 1.9 million US children suffer a sports- or recreation-related concussion annually.^2^ Numerous studies have documented the incidence of SRCs among high school and collegiate athletes. Many report female athletes to have higher SRC rates than males in comparable sports at both high school and collegiate levels.^3^, ^4^, ^5^, ^6^, ^7^, ^8^, ^9^, ^10^, ^11^, ^12^ These sex differences in SRC rates have been attributed to variations in a range of factors, including biomechanical, hormonal, cognitive, psychological, and sociocultural influences.^4^^,^^13^

Although substantial evidence exists regarding SRC in older adolescents and young adults, research on sex differences in SRC incidence among middle school (MS)-aged children (ages 10-13) remains limited, despite this age group representing the largest segment of organized sports participants in the US.^1^ Presently, it remains uncertain whether the sex differences observed in older athletes apply to early adolescents. Closing this knowledge gap is critical, because the significant physical, cognitive, and psychosocial changes that occur alongside emerging sex differences during early adolescence may require tailored preventive strategies and care considerations.^14^^,^^15^ Recognizing this gap, the Concussion in Sport Group, in its most recent consensus statement, called for greater representation of both younger and female athletes in SRC research.^16^ Therefore, the purpose of this study was to investigate potential sex differences in SRC rates among early adolescent children participating in sex-comparable MS sports. Building on previous research, we hypothesized that MS girls would have significantly higher concussion rates than their male counterparts.

## Methods

### Setting and Participants

Data were gathered among students (6th-8th grades) participating in sports at their respective MSs within a large, socioculturally diverse (70.1% minority; 40.9% economically disadvantaged) public school district in Virginia between the 2015 and 2022 school years (Table I).^17^ Between the 2015 and 2019 school years, 9 MS were included in the study. This number increased to 16 MS in 2019 and 17 MS in 2020. Children who participated in at least 1 of the following MS sports were included in the study: baseball, basketball, soccer, track and field, and softball. Owing to COVID-19, there were no sports (girls' soccer, softball, and boys' baseball, track and field) during the spring 2020 season or the 2020-2021 school year; therefore, no injury data were gathered during these periods. The Institutional Review Board of George Mason University (#717033-9) granted approval for this study and waived the requirement for informed consent and assent, because the research was retrospective.

**Table I:** Total MS student population demographics from 2015 to 2022 school years

Race	%	% Economically disadvantaged
American Indian or Alaska Native	0.2	0.1
Asian	9.0	3.5
Black, not of Hispanic origin	20.3	9.4
Hispanic	34.5	22.4
Native Hawaiian or Pacific Islander	0.2	0.0
Non-Hispanic, two or more races	5.9	1.5
White, not of Hispanic origin	29.9	4.0
Total	100.0	40.9

Estimates are based on fall enrollment data. Proportions calculated based on MS total student enrollment (n = 630,653) for study period. Ecumenically disadvantaged students met any one of the following: (1) eligible for free/reduced meals, or (2) received Temporary Assistance for Needy Families (TANF), or (3) eligible for Medicaid, or (4) identified as either Migrant, experiencing Homelessness, Foster, or Head Start.

### Procedures

As part of the ACHIEVES (Advancing Health Care Initiatives for Underserved Students) Project, certified athletic trainers (ATs) were embedded in MSs. ATs were present at all school-sponsored sports events and collected athlete exposure (AE) and injury data. All injury data were collected using the SportsWare electronic injury tracking software (version VI.99.4.1, Computer Sports Medicine, Stoughton, MA) as a part of daily clinical practice. ATs completed all initial sport-related injury documentation on the day of injury and could return to entries to update them as needed. Daily attendance was recorded by sport for all events and was used to calculate AE values.^18^

### Operational Definitions

#### SRC

Consistent with prior epidemiological reports and international consensus among athletic populations, a reportable SRC was defined as an injury that (1) occurred as a result of participation in a school-sponsored sport-related event, (2) required the athlete to be removed from activity after exhibiting signs, symptoms, or behaviors consistent with an SRC, (3) required care from an AT or physician, and (4) was clinically diagnosed as a SRC.^19^, ^20^, ^21^ In Virginia, licensed ATs are permitted to diagnose and manage concussions in accordance with the Code of Virginia and guidelines established by the Virginia Board of Education.^22^^,^^23^ Throughout the study, concussion policies and care protocols aligned with published consensus statements and Virginia legislation.^22^, ^23^, ^24^, ^25^ All MS ATs underwent annual training to ensure alignment of clinical care with current policy, legislation, and best practice. Additionally, ATs also routinely participated in quality assurance protocols to ensure consistent and accurate data collection.

#### Sex-comparable MS Sports

Consistent with prior methods, sex-comparable MS sports were those in which both male and female athletes competed in the same sport following similar rules, but they played on separate teams for males and females.^4^^,^^7^^,^^12^ Sex-comparable sports that were included in our study were MS (i) girls' softball and boys' baseball, (ii) girls' and boys' basketball, (iii) girls' and boys' soccer, (iv) and girls' and boys' track and field.^4^^,^^7^^,^^12^

#### AE

An AE in this study was defined as one athlete participating in one school-sponsored event (practice or competition) where there is a possibility of injury, regardless of the duration of participation.^18^

#### Event Type

The type of school-sponsored event where the SRC occurred was classified as practice or competition.

#### SRC Mechanism

The primary physical event that caused the SRC, was classified as either direct contact with a ball, player, surface, field equipment, or nonspecific contact. Nonspecific contact was defined as a confirmed direct contact mechanism that was not clearly specified by the injured athlete or eyewitness report. A mechanism was classified as unknown when the injured athlete and witnesses were unable to specify the SRC cause.^5^

### Statistical Analyses

SRCs associated with participation in eight sex-comparable MS sports were included for inferential statistical analyses. Frequencies and SRC injury rates (IR) were calculated per 1000 AEs. IR ratios (IRRs) compared SRC IRs between MS girls and boys participating in sex-comparable sports and by event type (competitions and practices). Injury proportion ratios were used to compare the distributions of SRC mechanisms by sex. IRRs and injury proportion ratios with a 95% CI that excluded 1.00 were considered statistically significant.^26^ All data were analyzed using R software (version 4.0.2, The R Foundation for Statistical Computing, Vienna, Austria).^26^

## Results

A total of 11,213 athletes (girls, n = 5647 [50.4%], aged 12.4 ± 1.0 year; boys, n = 5566 [49.6%], aged 12.6 ± 1.0 years) participated in the eight sex-comparable MS sports (329,269 AEs) during the study period. A total of 97 SRCs were diagnosed for an overall SRC rate of 0.29/1000 AEs (95% CI, 0.24-0.35), with most occurring during competition (n = 56, 58%; IR, 0.71/1000 AE; 95% CI, 0.52-0.89) followed by practice (n = 41 [42%]; IR, 0.16 (0.11-0.21). Overall, girls accounted for 68% (n = 66) of all SRC and had a higher rate than boys (girls IR, 0.41/1000 AE; 95% CI, 0.31-0.51; vs boys IR, 0.18/1000 AE; 95% CI, 0.12-0.25). The sport with the highest SRC incidence for both sexes was soccer (girls, 0.96/1000 AE; 95% CI, 0.62-1.30; boys, 0.38/1000 AE; 95% CI, 0.20-0.55). Table II provides information regarding SRC frequencies, proportions, AEs, and rates by sex, sport, and event type.

**Table II:** SRC frequencies, proportions, AEs, and rates (with 95% CIs) by sex, sport, and event type

Categories	Practice			Competition			Overall		
	No. (%)	AE (%)	IR/1000AE	No. (%)	AE (%)	IR/1000AE	No. (%)	AE (%)	IR/1000AE
Girls	29 (44)	120,693 (76)	0.18 (0.12-0.25)	37 (56)	38,908 (24)	0.23 (0.16-0.31)	66 (68)	159,601 (48)	0.41 (0.31-0.51)
Basketball	11 (44)	33,066 (71)	0.33 (0.14-0.53)	14 (56)	13,576 (56)	1.03 (0.49-1.57)	25 (26)	46,642 (14)	0.54 (0.33-0.75)
Softball	4 (50)	15,291 (74)	0.26 (0.01-0.52)	4 (50)	5,395 (26)	0.74 (0.01-1.47)	8 (8)	20,686 (6)	0.39 (0.12-0.65)
Soccer	13 (42)	23,328 (72)	0.56 (0.25-0.86)	18 (58)	8,966 (28)	2.01 (1.08-2.94)	31 (32)	32,294 (10)	0.96 (0.62-1.30)
Track and Field	1 (50)	49,008 (82)	0.02 (0.00-0.06)	1 (50)	10,971 (18)	0.09 (0.00-0.27)	2 (2)	59,979 (18)	0.03 (0.0-0.08)
Boys	12 (39)	129,325 (76)	0.07 (0.03-0.11)	19 (61)	40,343 (24)	0.11 (0.06-0.16)	31 (32)	169,668 (52)	0.18 (0.12-0.25)
Basketball	5 (56)	41,178 (74)	0.12 (0.01-0.23)	4 (44)	14,626 (26)	0.27 (0.01-0.54)	9 (9)	55,804 (17)	0.16 (0.06-0.27)
Baseball	2 (67)	17,936 (76)	0.11 (0.00-0.27)	1 (33)	5,657 (24)	0.18 (0.00-0.52)	3 (3)	23,593 (7)	0.13 (0.00-0.27)
Soccer	4 (22)	35,364 (74)	0.11 (0.00-0.22)	14 (78)	12,228 (26)	1.14 (0.55-1.74)	18 (2)	47,592 (14)	0.38 (0.20-0.55)
Track and Field	1 (100)	34,847 (82)	0.02 (0.00-0.07)	0 (0)	7,832 (18)	—	1 (1)	42,679 (13)	0.02 (0.00-0.07)
Total	41 (42)	250,018 (76)	0.16 (0.11-0.21)	56 (58)	79,251 (24)	0.71 (0.52-0.89)	97 (100)	329,269 (100)	0.29 (0.24-0.35)

*n*, SRC frequency; *IR*, SRC rate; —, not applicable.

### Sex Differences in SRC Rates

MS girls were overall 2.26 times more likely to sustain SRCs than their male counterparts (IRR, 2.26; 95% CI, 1.78-2.88). Moreover, girls had significantly higher SRC rates compared with boys in 3 of the 4 sex-comparable sports: basketball (IRR, 3.32), softball/baseball (IRR, 3.04), and soccer (IRR, 2.54). There was no sex difference in the overall rate of SRC in track and field (IRR, 0.71). Differential rates for comparable sports by sex are shown in Table III.

**Table III:** IRRs for SRC by sex-comparable sport and event type

Sex-comparable sport	IRRs (95% CI)		
	Competition	Practice	Overall
Basketball	3.77 (2.38-5.98)∗	2.74 (1.68-4.47)∗	3.32 (2.37-4.65)∗
Softball/baseball	4.19 (1.75-10.08)∗	2.35 (1.05-5.22)∗	3.04 (1.68-5.49)∗
Soccer	1.75 (1.24-2.48)∗	4.92 (3.06-7.92)∗	2.54 (1.92-3.36)∗
Track and field	—	0.71 (0.18-2.84)	1.42 (0.46-4.41)
Overall	2.07 (0.67-6.42)	2.57 (1.89-3.49)∗	2.26 (1.78-2.88)∗

—, not applicable.

Girls' sports events were referent groups for IRR calculations (ie, Readers should interpret sex comparisons within each column; for example, the Basketball competition values represent the IRRs comparing male and female basketball athletes).

∗ Statistically significant differential rates.

### Sex Differences by Event Type

In this study, both girls (n = 37 [56%]) and boys (n = 19 [61%]) sustained most of the SRCs during competition (n = 56 [58%]) (Table II). The sport with the highest competition-related SRC rate for both sexes was soccer (girls, 2.01/1000 AE; 95% CI, 1.08-2.94; boys, 1.14/1000 AE; 95% CI, 0.20-0.55). When examined across all sex-comparable MS sports, there was no significant difference in the competition-related SRC rates between girls and boys. However, significant differences in competition-related SRC rates were noted for individual sex-comparable sports: softball/baseball (IRR, 4.19), basketball (IRR, 3.77), and soccer (IRR, 1.75) (Table III). Our practice-related SRC findings revealed a notable difference between girls and boys. Girls were 2.57 times more likely to experience an SRC during practice than boys in sex-comparable MS sports (IRR, 2.57). The sport with the highest practice-related SRC rate for girls was soccer (0.56/1000 AE; 95% CI, 0.14-0.53), whereas for boys it was basketball (0.12/1000 AE; 95% CI, 0.01-0.23). Significant differences in practice-related SRC rates were observed in 3 of the 4 sex-comparable sports studied: notably, soccer practice had the greatest difference, with girls nearly 5 times more likely to sustain an SRC than boys (IRR, 4.92). This was followed by basketball (IRR, 2.74) and softball/baseball (IRR, 2.35). There was no significant difference in the practice-related SRC rate between girls and boys in track and field (IRR, 0.71). Figure provides a comparison of SRC rates by sex-comparable sport and event type.

**Figure:**
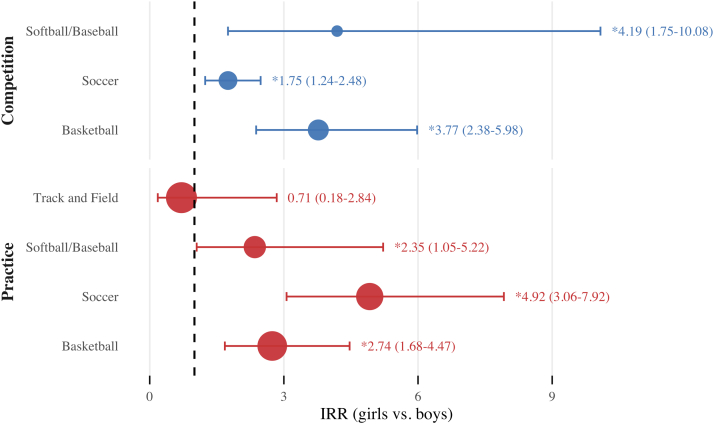
Comparison of competition and practice SRC rates in MS girls and boys participating in sex-comparable sports, with girls' sports events as the reference group. Differential rates are expressed as IRRs, which compare girls' sports with boys' sports, along with 95% CIs. The size of the point estimate marker is proportional to total AEs by sport and event type. The dashed vertical line represents a null value of 1.00. Effect estimates with horizontal bars (95% CIs) that do not touch the vertical line are statistically significant.

### Sex Differences by Mechanism

SRC mechanisms by sex-comparable MS sports are presented in Table IV. Among girls, ball contact accounted for a majority of SRCs (n = 22 [33.3%]), followed by player contact (n = 19 [28.8%]). For boys, player contact accounted for most SRCs (n = 10 [32.3%]), followed by ball contact (n = 9 [29.0%]). Injury proportion ratios revealed no significant difference in the relative distribution of SRCs caused by player contact mechanisms by sex.

**Table IV:** Frequencies and proportions of SRC mechanisms by sex-comparable sport

Categories	SRC mechanisms						
	Player	Surface	Equipment	Ball	Nonspecific	Unknown	Total
Girls'	19 (29)	7 (11)	3 (5)	22 (33)	14 (21)	1 (2)	66 (100)
Basketball	10 (40)	2 (8)	1 (4)	8 (32)	4 (16)	0 (0)	25 (100)
Softball	0 (0)	0 (0)	0 (0)	7 (88)	1 (13)	0 (0)	8 (100)
Soccer	8 (26)	5 (16)	1 (3)	7 (23)	9 (29)	1 (3.2)	31 (100)
Track and field	1 (50)	0 (0)	1 (50)	0 (0)	0 (0)	0 (0)	2 (100)
Boys'	10 (32)	6 (19)	0 (0)	9 (29)	4 (13)	2 (7)	31 (100)
Basketball	4 (44)	4 (44.4)	0 (0)	0 (0)	0 (0)	1 (11.1)	9 (100)
Baseball	0 (0)	0 (0)	0 (0)	3 (100)	0 (0)	0 (0)	3 (100)
Soccer	6 (33)	1 (6)	0 (0)	6 (33)	4 (22)	1 (5.6)	18 (100)
Track and field	0 (0)	1 (100)	0 (0)	0 (0)	0 (0)	0 (0)	1 (100)
Overall	29 (30)	12 (12)	3 (3)	31 (32)	18 (19)	3 (3)	97 (100)
IPR (95% CI)	0.89 (0.69-1.09)	—	—	—	—	—	

—, not applicable; *IPR*, injury proportion ratio.

Values are number (%) and IPRs. IPRs excluded because of SRC counts <10.

## Discussion

This study offers new insights into sex differences in SRC incidence among early adolescents, a population that has been under-represented in SRC research. Consistent with patterns reported among older athletes, we observed that across sex-comparable MS sports, girls had approximately twofold higher SRC rates than boys (except track and field).^3^^,^^4^^,^^7^^,^^9^^,^^12^ These data indicate that sex-based differences in SRC risk are already evident by early adolescence, emphasizing the importance of directing greater attention toward improving SRC prevention and clinical care for MS-age female athletes.

### Overall Sex Differences in MS SRC

Early adolescent girls in this study had significantly higher SRC rates than boys in 3 of 4 sex-comparable MS sports (basketball, softball/baseball, and soccer), aligning with prior findings in older high school and collegiate cohorts.^3^, ^4^, ^5^^,^^9^^,^^12^^,^^27^, ^28^, ^29^ However, our findings differ from Beachy and Rauh's study of MS athletes, which reported overall higher SRC rates among boys, a difference likely owing to their inclusion of football, a mainly male collision sport.^30^

Examining SRC mechanisms is important for understanding emerging sex-specific patterns. Injury proportion ratios showed no significant sex differences in mechanism distributions among MS girls and boys. Player and ball contact accounted for the majority of SRCs in both MS girls and boys, whereas surface, equipment, and nonspecific mechanisms were less common causes. This pattern contrasts with research at the high school level, where boys more frequently sustained SRCs from player contact and girls more often from ball or equipment-related mechanisms.^5^^,^^6^ Thus, suggesting that the divergence of SRC mechanisms observed in older athletes may emerge after MS, potentially reflecting later biopsychosocial and sport-specific changes.

### SRC Sex Differences in Contact Sports

MS girls involved in contact sports, specifically soccer (0.96/1000 AEs) and basketball (0.54/1000 AEs), demonstrated the highest SRC rates in this study. These findings are consistent with a systematic review and meta-analysis by Ernst et al, which identified soccer and basketball as the high school girls' sports with the highest SRC rates (0.72 and 0.43/1000 AEs, respectively).^31^ In our MS cohort, the SRC rate for girls' soccer was 2.5 times higher than for boys' soccer (0.96/1000 AEs vs 0.39/1000 AEs), and for girls' basketball, it was 2.7 times higher than for boys' basketball (0.54/1000 AEs vs 0.20/1000 AEs). Similar sex differences have been documented in high school sports. Kerr et al reported girls' soccer and basketball SRC rates were approximately 2.3 times higher than those of boys' (0.82/1000 AEs vs 0.36/1000 AEs and 0.48/1000 AEs vs 0.21/1000 AEs, respectively).^5^ Lincoln et al similarly reported that girls' soccer rates were nearly double those of boys' (0.35/1000 AEs vs 0.17/1000 AEs), and basketball rates were 1.6 times higher (0.16/1000 AEs vs 0.10/1000 AEs).^3^ Recent national data from Chandran et al support this trend, showing SRC rates for girls' soccer 1.8 times higher than boys' soccer (0.61/1000 AEs vs 0.33/1000 AEs) and girls' basketball 2.2 times higher than boys' basketball (0.40/1000 AEs vs 0.18/1000 AEs).^6^

Sex differences in MS contact sport SRC rates were also evident by event type. In soccer, the girls' competition SRC rate (2.01/1000 AEs) was 1.76 times higher than the boys' (1.14/1000 AEs), and the girls' practice SRC rate (0.56/1000 AEs) was more than 5 times higher than the boys' (0.11/1000 AEs). In basketball, girls' competition SRC rate (1.03/1000 AEs) was 3.8 times higher than boys' (0.27/1000 AEs), and the practice SRC rate (0.33/1000 AEs) was 2.7 times higher than boys' (0.12/1000 AEs). These findings align with previous studies of high school athletes, which show girls' SRC rates are 1.5-2.0 times higher than boys' across both practice and competition in soccer and basketball.^3^^,^^5^^,^^6^

### SRC Sex Differences in Limited Contact and Noncontact Sports

In limited-contact sports, MS girls also demonstrated higher SRC rates than boys. A previous study of MS athletes from the 2015-2016 season reported SRC rates of 0.68 and 0.57/1000 AEs in softball and baseball, respectively. However, the low SRC count prevented meaningful sex comparisons.^18^ By contrast, in our multiyear study, the overall SRC rate for girls' softball was 3 times higher than boys' baseball (0.39/1000 AEs vs 0.13/1000 AEs). Significant sex differences in MS limited-contact sport SRC rates were also observed across event types. In girls' softball, the practice SRC rate was 2.4 times higher than that of boys' baseball (0.26/1000 AEs vs 0.11/1000 AEs). We observed the SRC rate differential between MS girls' softball and boys' baseball to be most pronounced during games, where girls' softball had an SRC rate that was 4.2 times higher than boys' baseball (0.74/1000 AEs vs 0.18/1000 AEs). These findings align with research at the high-school level, where SRC rates in girls' softball also significantly exceed those of boys' baseball (IRR ≈ 2.2-3.1).^5^^,^^6^

Regarding SRC mechanisms, ball contact was the most common mechanism of SRC in both MS girls' softball (88%) and boys' baseball (100%). This finding aligns with high school softball data, which reported that impacts from equipment, particularly ball contact, were the primary mechanism of SRCs.^5^^,^^6^^,^^32^ However, for high school baseball, reports suggest SRC mechanisms are more evenly split between ball contact and player contact.^5^^,^^32^ A possible explanation for these sex differences in SRC rates may lie in game dynamics unique to MS softball, such as the shorter pitching distance and ball trajectory, which may reduce players' time to react and avoid ball contacts and, as a result, increase SRC risk.^32^

Track and field, a noncontact sport, had the lowest SRC rates and was also the only sex-comparable sport where no significant differences in SRC rates were observed between MS girls and boys. Although the pattern aligns with previous research on SRC rates in high school and college athletes, these results should be interpreted cautiously, as only 3 SRC cases occurred in MS track and field, substantially limiting our ability to detect sex differences.^5^^,^^6^^,^^20^

### Socioecological Contributors to Sex Differences in MS SRC

In this MS cohort, early adolescent girls demonstrated significantly higher SRC rates than boys overall and during both competition and practice in soccer, basketball, and softball/baseball. Although similar sex differences have been documented in older athletic populations, the rate differential between MS girls and boys was larger than that reported in high school and college athletes.^3^^,^^5^^,^^6^^,^^33^ These differences likely reflect multiple, intersecting influences operating across socioecological levels.

At the individual level, several biological mechanisms contribute to MS girls' earlier pubertal onset, including interacting biomechanical, hormonal, and neurodevelopmental factors. Anatomically, females have smaller head-neck segment mass and lower cervical muscle strength, which may increase head acceleration impact. For example, lower average cervical muscle strength and smaller head-neck segment mass among females may increase susceptibility to biomechanical forces that cause SRC.^34^^,^^35^ Additionally, reports suggest that girls may have heightened interoceptive awareness of their internal states (ie, more apt to recognize and acknowledge symptoms), which affects both how they express symptoms and their likelihood of reporting them.^14^^,^^15^^,^^36^

At the school, team, and interpersonal levels, contextual and environmental factors shape both SRC exposure and reporting. Girls' sports environments have been characterized by greater variation in skill, which may increase the likelihood of unexpected impacts leading to SRC.^37^^,^^38^ Reporting patterns likewise differ by sex: girls are more likely to disclose symptoms to teachers or school nurses, whereas boys more often report to coaches and may normalize “playing through” injuries, resulting in under-reporting.^39^^,^^40^ Additionally, variations in coaching expertise, rule enforcement, and availability or use of protective equipment across girls' sports may further contribute to the higher SRC rates observed among MS girls compared with boys.^31^^,^^41^^,^^42^

At the organizational and policy levels, structural factors also influence observed sex disparities in SRC. Girls' sports have less access to onsite medical care (eg, ATs), greater variability in SRC policy implementation, and unequal access to specialized concussion-related medical care. Differences in school-based reporting and injury surveillance protocols may further influence how SRC events are identified and documented. Evidence also indicates that girls are less likely than boys to be promptly removed from play after a suspected concussive event, which can influence both symptom reporting and care-seeking behaviors.^43^, ^44^, ^45^

Collectively, these findings underscore the need to identify and more fully understand the multilevel biopsychosocial determinants of SRC and to promote brain health among female athletes during the developmentally sensitive period of early adolescence. Future research should use longitudinal, mixed-methods approaches to clarify the relative contributions of biological vulnerability, reporting behaviors, and systemic factors to the sex differences in SRC incidence observed among MS athletes.

### Strengths and Limitations

Data were derived from a convenience sample of 17 public MS staffed with certified ATs, enhancing the reliability of record keeping and diagnostic consistency. Therefore, our findings may not be generalizable to all other MS-aged athletes, particularly those without ATs. Furthermore, our study design categorized sex as male or female. Future research should incorporate gender identity and consider social determinants of health. Although ATs followed a standardized concussion assessment protocol, as with all studies, clinical judgment may have introduced variability. A key strength of this study was the diversity of the participating school division, as well as the model of embedded ATs, which may offer a more accurate representation of the true SRC incidence among pediatric athletes.

## Conclusions

This study provides valuable insights into sex-based differences in SRC rates among early adolescents participating in MS sports, a population previously under-represented in SRC research. MS girls had an SRC rate more than twice that of boys in sex-comparable sports, with significantly higher rates during both competition and practice. Although comparable sex differences have been observed in older athletic populations, the disparity between MS girls and boys was larger than reported in high school and college-level athletes. Despite this increased burden, evidence to guide female-specific SRC prevention remains limited. Only a small fraction of existing studies include female athletes aged <18 years, and even fewer specifically target strategies to reduce concussion risk among early adolescent girls. These findings demonstrate that sex-based differences in SRC risk are evident by early adolescence, underscoring the need for rigorous SRC research to identify and inform prevention strategies that reflect the developmental characteristics and sport environments of MS girls.

## Declaration of Generative AI and AI-assisted Technologies in the Writing Process

During the preparation of this manuscript, Grammarly (San Francisco, CA) was used to assist with grammar, spelling, and punctuation checks, as well as to improve the clarity and concision of the text. All authors have reviewed and approved the final version of the manuscript and take full responsibility for its content.

## CRediT authorship contribution statement

**Samantha L. Hacherl:** Writing – original draft, Visualization, Methodology, Formal analysis, Data curation, Conceptualization. **Daniel C. Herman:** Writing – review & editing, Methodology, Formal analysis. **Joel R. Martin:** Writing – review & editing, Methodology, Formal analysis. **Patricia M. Kelshaw:** Writing – review & editing, Visualization, Methodology, Formal analysis. **Sanja Avramovic:** Writing – review & editing, Methodology, Formal analysis, Data curation. **Shane V. Caswell:** Writing – review & editing, Supervision, Project administration, Methodology, Funding acquisition, Formal analysis, Data curation, Conceptualization.

## funding

This work was funded in part through CDC Cooperative Agreement # NU17CE010060, CDC Block Grant # NB01PW000078, and HRSA Block Grant # B0454582 in partnership with the Virginia Department of Health.

## Declaration of Competing Interest

S.C. reports a relationship with Virginia Department of Health that includes funding grants. S.C. reports a relationship with Centers for Disease Control and Prevention that includes funding grants. S.C. reports a relationship with Prince William County Public Schools that includes funding grants. The authors received compensation for contributing to the Virginia Concussion Initiative, for which S.C. receives grant funding from the Virginia Department of Health and the Centers for Disease Control and Prevention. This funding was not related to any aspect of this manuscript but is associated with the topic of SRC (D.H., J.K., S.A.). If there are other authors, they declare that they have no known competing financial interests or personal relationships that could have appeared to influence the work reported in this paper.
